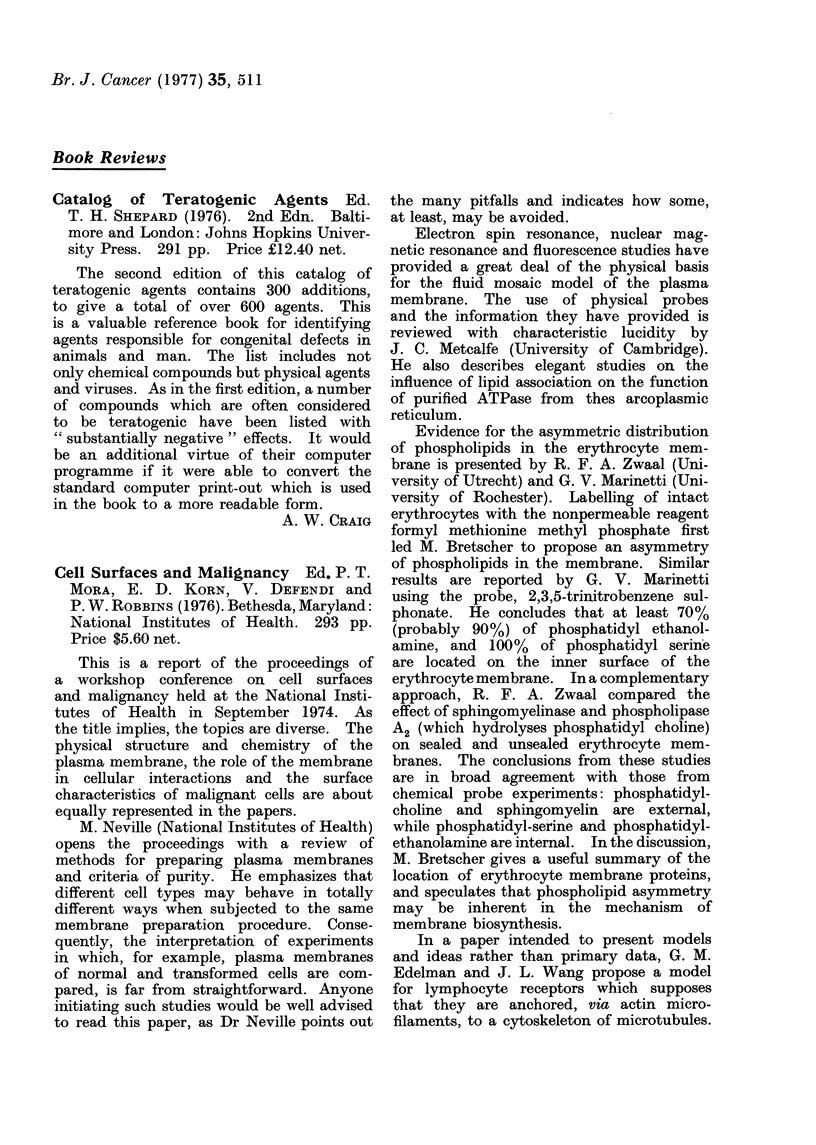# Catalog of Teratogenic Agents

**Published:** 1977-04

**Authors:** A. W. Craig


*Br. J. Cancer* (1977) **35**, 511

### *Book Reviews*

**Catalog of Teratogenic Agents** Ed. T. H. SHEPARD (1976). 2nd Edn. Baltimore and London: Johns Hopkins University Press. 291 pp. Price £12.40 net.

The second edition of this catalog of teratogenic agents contains 300 additions, to give a total of over 600 agents. This is a valuable reference book for identifying agents responsible for congenital defects in animals and man. The list includes not only chemical compounds but physical agents and viruses. As in the first edition, a number of compounds which are often considered to be teratogenic have been listed with " substantially negative " effects. It would be an additional virtue of their computer programme if it were able to convert the standard computer print-out which is used in the book to a more readable form.

A. W. CRAIG